# The Traumatic Tube: Bleeding Rectal Ulcer Caused by Flexi-Seal Device

**DOI:** 10.1155/2017/5278971

**Published:** 2017-10-04

**Authors:** Abhinav Tiwari, Himani Sharma, Khola Qamar, Yaseen Alastal, Thomas Sodeman, Ali Nawras

**Affiliations:** ^1^Department of Internal Medicine, University of Toledo Medical Center, Toledo, OH, USA; ^2^Department of Gastroenterology, University of Toledo Medical Center, Toledo, OH, USA

## Abstract

Diarrhea and fecal incontinence are common in critically ill patients and present a challenging problem in patient management. The Flexi-Seal® Fecal Management System is a device to divert the stools away from the patient, thus improving the care to patients with fecal incontinence. There have been only few case reports describing the complications with the use of this device. Here, we present a case of a 77-year-old woman who was admitted due to massive hematochezia while on anticoagulation. She was found to have a large rectal ulcer caused by the Flexi-Seal device, used during the last hospital stay for fecal incontinence. Flexi-Seal device can be effective for the management of incontinence; however, caution should be exercised during handling and pressure from the retention balloon should be relieved periodically.

## 1. Introduction

Fecal incontinence is not uncommon in acutely ill hospitalized patients, occurring in up to 33% of critically ill patients often in association with diarrhea [1]. The etiology of diarrhea or fecal incontinence, in critically ill patients, is often unknown and can be multifactorial. Managing diarrhea in such patients presents a challenging problem in patient management as it can lead to breakdown of skin, ulceration, and infection [2]. Flexi-Seal Fecal Management system (FSFMS) (ConvaTec Professional Services) was designed to provide a safe and effective diversion of liquid stool away from the skin and has been adopted by many intensive care units (ICUs). These devices reduce the need for labor-intensive nursing care, decrease the risk of perianal skin breakdown, and decrease transmission of fecal microbiota. These devices consist of a soft silicon catheter and a retention balloon that is inflated with 45 ml of saline or water. This balloon is inflated in the rectum to form a seal that prevents seepage of fecal contents around it. These devices are often believed to be safe and effective, and some studies have demonstrated their successful use in patients with diarrhea [3]. However, there have been few cases in the literature describing the adverse effects from the pressure necrosis or traumatic removal of such devices. Such complications include rectal ampullary ulcers, rectal fissures, and fatal bleeding due to rectal trauma. Although limited, such reports suggest that these devices may have a higher morbidity risk than previously believed. Here, we present a case of a 77-year-old woman who developed hematochezia while on anticoagulation. Further investigation revealed bleeding rectal ulcer secondary to the recent use of the Flexi-Seal system.

## 2. Case Report

**Figure 1:**
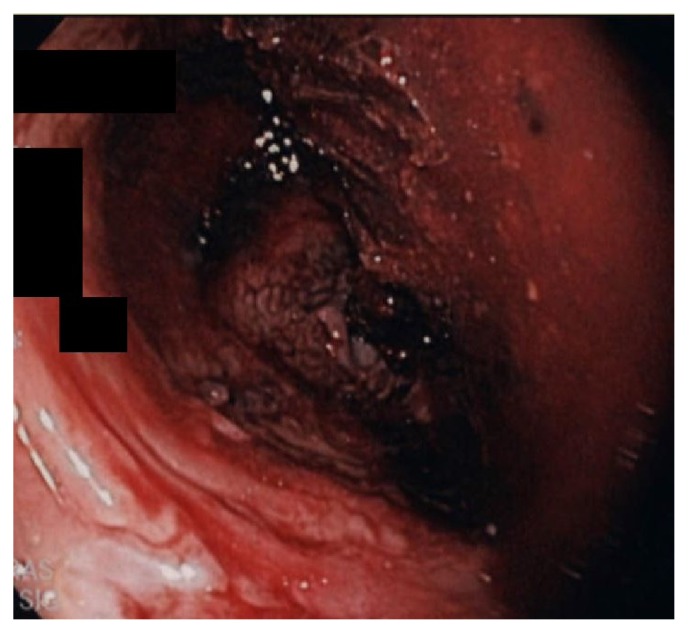
Flexible sigmoidoscopy showing a circumferential rectal ulcer with erythematous mucosa.

**Figure 2:**
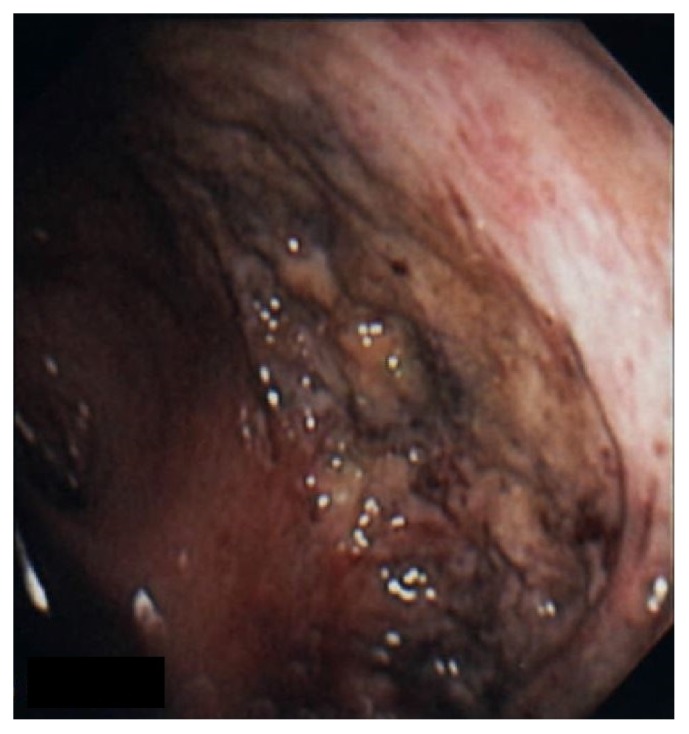
Flexible sigmoidoscopy showing a part of circumferential ulcer in the rectum.

A 77-year-old female with a history of interstitial lung disease was admitted to ICU due to multiple episodes of lower gastrointestinal bleeding. Vital signs revealed a blood pressure of 98/68 mm Hg, heart rate of 102 beats per minute, respiratory rate of 18/min, and temperature of 97.5 degrees Fahrenheit. Rectal examination was unremarkable except for bright red blood on the examining finger. Laboratory workup revealed a WBC count of 8.6 × 109/L, hemoglobin level of 8.9 g/dL, and platelet count of 250,000/*μ*L. Seven days before the current admission, she was discharged after an extended stay in ICU for respiratory failure requiring intubation and mechanical ventilation. She was diagnosed with bilateral lower extremity deep vein thrombosis and was discharged on apixaban. Hemoglobin level was 10.3 g/dL at the time of discharge. During the current hospitalization, apixaban was held, and the patient was given intravenous fluids. Gastroenterology consultation was obtained, and an esophagogastroduodenoscopy (EGD) was performed to rule out rapid upper gastrointestinal (GI) bleeding. EGD was unremarkable with no evidence of fresh or old blood until the second part of the duodenum. Flexible sigmoidoscopy showed some retained stool in the rectum. However, there was a large area of ulceration and erythema involving most of the circumference of the rectal wall (Figures 1 and 2). This ulcerated area extended up to 15 cm from the anal verge. The scope was advanced up to 35 cm from the anal verge and rest of the colonic mucosa was normal. Multiple biopsies taken from the ulcer edge revealed necrotic epithelium with no evidence of dysplasia or malignancy. Chart review revealed that the patient had Flexi-Seal system placed for low-volume diarrhea during the last hospital stay. Flexi-Seal was placed for a total of 9 days and removed after stool output slowed down. Otherwise, there was no history of rectal trauma or rectal surgery in the past.

Bleeding ceased after anticoagulation was discontinued and an inferior vena cava (IVC) filter was placed to prevent pulmonary embolism. The patient was discharged without anticoagulation. There was no subsequent episode of GI bleeding.

## 3. Discussion

Diarrhea and fecal incontinence are a widespread problem in critically ill patients [4–6]. Critical illness is by itself a risk factor for diarrhea, while other factors include physiological stress, medications (magnesium, laxatives), lack of fibers in enteral feed, and alteration in intestinal flora due to antibiotic use [4, 7]. Diarrhea can compromise the management of the patient and cause considerable morbidity due to skin breakdown and infection. Recently, the use of indwelling intrarectal fecal collection systems has gained popularity because of their effectiveness in diverting fecal material away from the patient. The Flexi-Seal device (FMS; ConvaTec, Division of E.R. Squibb & Sons, LLC, Princeton, NJ) is the most widely used device designed to be used continuously for up to 30 days.

The first report describing the potential adverse effects of the Flexi-Seal system was a prospective, multicenter, clinical study involving 42 patients [3]. This study reported 2 cases of generalized skin breakdown and one case of rectal bleeding resulting from pressure ulceration. Subsequently, there have been a handful of reports of adverse effects arising from the use of such device. There are 9 case reports in the literature reporting 13 patients who had lower GI bleeding secondary to either an ulcer or laceration secondary to use of Flexi-Seal device.

Page et al. (2008) reported the case of a 65-year-old man who developed severe bleeding from the rectal mucosa due to a laceration, 6 cm from the anal verge, which was sutured. This laceration was attributed to the use of the Flexi-Seal system for six days [8]. Bright et al. [9] reported lower GI bleeding in a 79-year-old man 11 days after insertion of a Flexi-Seal device. Colonoscopy findings at the time were suggestive of pressure necrosis in the rectal area. Mesenteric angiography showed extravasation of contrast material from a branch of the superior rectal artery, and coil embolization was successfully performed. Sparks et al. (2010) reported 3 cases of massive GI bleeding associated with the use of the Flexi-Seal device [10]. The first patient, a 72-year-old woman, presented with two episodes of lower GI bleeding due to a rectal laceration 4 cm proximal to the dentate line. The laceration was sutured after the second episode of bleeding. The second patient was a 54-year-old woman on warfarin who also presented with lower GI bleeding secondary to a tear of the anterior rectal wall 3 cm from the anal verge. The tear was treated with direct pressure, application of thrombin, and suture ligation. The third patient was a 59-year-old man on warfarin who was admitted due to a single episode of GI bleeding secondary to a mucosal ulcer in the proximal part of the anal canal. No treatment other than reversal of anticoagulation was required. Monge et al. (2011) reported two patients with rectal hemorrhage associated with the Flexi-Seal device [11]. The first patient was a 71-year-old man on prophylactic dose enoxaparin who presented with rectal hemorrhage 25 days after insertion of the device. Endoscopy revealed a mucosal ulcer in the distal part of the rectum. The second patient was a 67-year-old woman who had two episodes of rectal bleeding secondary to a mucosal ulcer in the posterior distal part of the rectum. Walsh and Sanders (2010) reported a case of fatal hemorrhage arising from a rectal ulcer caused by rectal tube [12]. The patient died despite clipping, banding, and injecting the lesion with epinephrine.

Reynolds and van Haren (2012) reported a case in which a 50-year-old man on heparin developed lower GI bleeding five days after insertion of a Flexi-Seal device [13]. Endoscopy revealed a mucosal ulcer in the distal part of the rectum 10 cm from the anal verge. The lesion was cauterized, injected with epinephrine, and suture-ligated. Mulhall and Jindal (2013) reported a case of a 58-year-old man with lower GI bleeding 13 days after insertion of the Flexi-Seal device [14]. Angiography revealed bleeding from a medial branch of the superior rectal artery which was then embolized. Eleven days later, the patient developed another episode of massive rectal bleeding and colonoscopy showed a posterior rectal laceration that was not actively bleeding and an anterior rectal ulcer with active hemorrhage. Hemostasis of the ulcer was achieved by suture ligation and application of topical thrombin. Popek and Senagore (2013) reported two cases of Flexi-Seal device-related complication [15]. The first case involved a 60-year-old woman who developed bright red blood per rectum from a bleeding rectal ulcer for which a clip was placed. Two weeks later, the patient again developed rectal bleeding, and an angiogram identified a bleeding vessel in the rectum, which was suture-ligated. The second patient was a 39-year-old patient admitted for sepsis and multiple organ failure. Three weeks after placement of the Flexi-Seal device, he began having bright red blood per rectum. Flexible sigmoidoscopy demonstrated a pulsatile vessel in the rectum, and the patient was taken to the operating room for suture ligation of the vessel. Shaker et al. (2014) reported a case of a 75-year-old female with atrial fibrillation on warfarin [16]. She developed lower GI bleeding 12 days after insertion of the Flexi-Seal device. CT angiogram showed that the bleeding was originating from the distal rectum. Flexible sigmoidoscopy showed severe ulceration involving the whole circumference of the rectum and evidence of active bleeding from a small artery. Hemostasis was achieved via argon-beam photocoagulation, a hemostatic stitch to the bleeding point, and packing of the rectum with alginate ribbon.

Whiteley et al. published a retrospective study analyzing the complications of Flexi-Seal in acute care setting [17]. The records of 50 patients who had a total of 69 study FMS inserted (mean: 1.4 FMS) were included in the study. Most patients (37, 74%) experienced no complications; 7 (14%) had their retention balloon overinflated but suffered no injury to the rectal mucosa; 4 (8%) experienced temporary anal atony; and 2 (4%) suffered excessive leak of stool around the device.

The pathophysiology of rectal ulceration or laceration due to the Flexi-Seal device is due to the pressure exerted by the retention balloon that, when inflated with saline, allows the catheter to remain anchored in the rectal ampulla. Build-up of semisolid fecal material above the level of the balloon may also contribute towards pressure necrosis, a mechanism similar to stercoral perforation caused by fecal impaction in severely constipated patients [18]. Therefore, the retention balloon should be periodically deflated to allow the fecal build-up to flow around the tubing and to release pressure on the mucosal wall.

This is the second case report in the literature of circumferential rectal ulceration secondary to a Flexi-Seal device. Our patient was on anticoagulation, which might have triggered bleeding from the rectal ulcer. However, as described above, there have been cases where the patient had substantial lower GI bleeding without anticoagulation.

In conclusion, Flexi-Seal is a useful device for controlling fecal incontinence. However, it is important to exercise caution with prolonged use of this device. Patients on anticoagulation should be closely monitored for any signs of lower gastrointestinal hemorrhage. The continued need for the utilization of this device should be assessed daily, and a low threshold for removal should be employed.
